# A Meta-Synthesis on Clients’ Experience with Telerehabilitation

**DOI:** 10.3390/healthcare14060717

**Published:** 2026-03-11

**Authors:** Yughdtheswari Muniandy, Karmegam Karuppiah, Muhammad Hibatullah Romli, Rajkumar Krishnan Vasanthi, Nina Fatma Ali, Thenmoly Subramaniam, Jayesh Chandran, Haidzir Manaf, Hernan Cortez Labao

**Affiliations:** 1 Department of Rehabilitation Medicine, Faculty of Medicine and Health Sciences, Universiti Putra Malaysia, Serdang 43400, Malaysia; eshwari_physiorehab@yahoo.com (Y.M.);; 2 Faculty of Health and Life Sciences, INTI International University, Bandar Baru Nilai 71800, Malaysia; 3 Department of Environmental and Occupational Health, Faculty of Medicine and Health Sciences, Universiti Putra Malaysia, Serdang 43400, Malaysia; 4 Nitte Institute of Physiotherapy (NIPT), Nitte (Deemed to be University), Mangalore 575018, Karnataka, India; 5 Centre for Physiotherapy Studies, Faculty of Health Sciences, Universiti Teknologi MARA, Puncak Alam Campus, Bandar Puncak Alam 42300, Malaysia; 6 College of Physical Therapy, Our Lady of Fatima University, Valenzuela City 1440, Philippines

**Keywords:** telerehabilitation, digital health, rehabilitation, patient experience, qualitative synthesis, public health

## Abstract

**Background:** Telerehabilitation (TR) enables equitable access to therapeutic care over a distance; however, there is still a level of uncertainty about its efficiency in comparison with the traditional methods. Perception of patients has been inconclusive with aspects of technology use, quality care provision, and the satisfaction of patients with TR services being raised as issues to be investigated further. A systematic synthesis of patient experience is needed to establish TR’s general implications on health and well-being. Hence, the aim of this study is to review perceptions and experiences of clients on telerehabilitation. **Methods:** A systematic review of qualitative studies was conducted using eight electronic databases from inception to February 2026. Data were synthesized and the Critical Appraisal Skills Programme (CASP) tool was employed to assess quality of included studies. **Results:** The search strategy identified a total of 1667 articles and after removing duplicates and excluding articles based on title and abstract, and full text screening, 26 studies were included. Four major themes were developed: enhanced accessibility and reduced burden, perceived efficiency and continuity of care, therapeutic relationship and social presence, and digital and contextual challenges. **Conclusions:** Telerehabilitation reshapes rehabilitation delivery rather than merely adding technology. Its effectiveness depends on alignment among patient capability, clinical needs, and organizational support. When supported by adequate infrastructure and relational adaptation, it can enhance access and continuity and contribute to inclusive health delivery. However, if digital and contextual barriers remain unaddressed, telerehabilitation may inadvertently reinforce existing inequities.

## 1. Introduction

Telerehabilitation (TR) is a subfield of telemedicine service in which modern telecommunication techniques are used to support the delivery of rehabilitation at a distance [1]. This evolutionary paradigm increases the level of accessibility and overcomes the difficulties faced by people living in geographically remote areas or those with mobility limitations [2,3]. The financial sustainability of TR is a crucial advantage, as it reduces both the economic strain on the patient and health care systems [4,5]. Additionally, TR fosters greater patient engagement and autonomy, which is considered the key to effective rehabilitation [1]. There is an emerging body of evidence that TR is viable and effective in a wide range of conditions in diverse demographic groups, including both geriatric and pediatric populations [5,6,7,8,9]. Studies have also investigated awareness and utilization of TR, which has raised the positive advantages of using it and the challenges facing its implementation.

The field of telerehabilitation allows clinicians to follow the patients using the synchronous or asynchronous methods [1]. Synchronous TR refers to live interactions between clinicians and patients by means of video calling using a smartphone, tablet or computer connected camera allowing for immediate feedback and support [10]. In contrast, asynchronous TR is not a real-time approach, instead, it involves store-and-forward technologies, where patients engage with rehabilitation materials at their own pace [10,11]. Both approaches offer distinct advantages and challenges, with synchronous methods providing immediate interaction and support, while asynchronous options allow for greater flexibility and self-paced learning [3,12]. The technological modalities that have been implemented to provide rehabilitation services include video conferencing and chat messaging, as well as patient portals, making the process of communication easier and enriching the experience of patients with heterogeneous needs and preferences [13]. The effective operation of such digital platforms often relies on interoperable health information systems and standardized data exchange frameworks that facilitate secure communication and continuity of care across healthcare services [14].

Regardless of the ever-growing body of evidence supporting TR, it is necessary to note that continuous study is needed to completely clarify its long-term impacts on patient outcomes and health care provision [15]. Understanding the clinicians’ difficult ties in utilizing TR is equally important. Recent research highlights the need to train clinicians thoroughly and equip them to embrace TR within their expected working load boundaries and provide adequate access to technical resources [16]. Likewise, the perceptions and experiences of the patients with TR should be taken into consideration since they may have a direct impact on the knowledge, attitudes of the clinicians, and perceived appropriateness of TR implementation. Some patients may find the use of technology in healthcare intimidating or difficult, and those with little knowledge of TR services may have concerns about the quality of care delivered [17,18,19]. Understanding these perspectives is essential for developing strategies that enhance the overall effectiveness, acceptance, and sustainability of TR in clinical practice.

In line with this, the examination of patient experiences with TR provides detailed information on the perception of patients and possible barriers to successful implementation. By gathering insights from patients, healthcare providers can tailor TR services better to meet their needs and preferences, ultimately improving satisfaction and outcomes [20].

In accordance with that, numbers of qualitative studies have been conducted that provide insight into patients’ perception of TR and these have shown mixed results. Furthermore, these studies have not been systematically synthesized. A comprehensive synthesis of qualitative evidence is therefore necessary to clarify patient experiences, identify common themes, and inform future service improvements. Hence, this study aims to explore the diverse experiences of patients using TR, understand their perceptions and identify potential barriers to its successful implementation.

## 2. Materials and Methods

This meta-synthesis was conducted in accordance with the methodological framework delineated for the synthesis of qualitative research which provides a comprehensive and systematic approach [21]. The review protocol has been officially registered with the International Platform of Registered Systematic Review and Meta-analysis Protocols (INPLASY) under the identifier INPLASY PROTOCOL 202180047. The corresponding Digital Object Identifier (DOI) for the registered protocol is https://doi.org/10.37766/inplasy2021.8.0047 (accessed on 21 February 2026)The formal registration of the protocol ensured that the meta-synthesis adhered to a predetermined methodological framework, thereby augmenting both the transparency and rigor of the synthesis process.

### 2.1. Defining the Research Question

The usage of TR has a remarkable increase which gives rise to exploring the perception of clients using TR. For establishing key elements of the review question, we use a PICO (Population, Intervention, Comparison, and outcome) framework [22]. Although PICO is commonly applied in quantitative research, it was adapted in this review to structure the population and intervention components, while experiential outcomes were explored through interpretive thematic synthesis. The population in the review comprises clients including patients and caregivers who undergo rehabilitation using TR services, the intervention involves physiotherapy, occupational therapy, speech or medical rehabilitation, comparison with conventional rehabilitation (face to face) and outcome includes perception and experience of clients. The complete review question was “What is the experience and perception of the client using telerehabilitation?

### 2.2. Selection Criteria

To be considered in this review, each study has to meet the following inclusion criteria (1) qualitative study; (2) focus on the experience of clients in using teleconference application for rehabilitation; (3) the study is exploring rehabilitation-related topics (i.e., rehab medicine, occupational therapist, physiotherapist and speech pathologist); (4) clients including patients and caregivers. Meanwhile, the exclusion criteria are (1) studies investigate on mobile apps; (2) non-English articles; (3) studies in the gray literature (thesis, book, and conference); (4) studies involve monitoring and assessment (as our focus is to understand the merit and demerits using rehabilitation services).

### 2.3. Search Strategy

An extensive systematic literature search was conducted from January 2010 to 6 March 2025 using eight electronic bibliographic databases; Academic Search Complete, CINAHL, Health Business Elite, MEDLINE, Psychology, and Behavioral Sciences Collection, SPORTDiscus, Scopus, ASEAN Citation Index (ACI) with the full-text original article. Three different strings of keywords were used: (telerehabilitation OR “tele-rehabilitation” OR telehealth OR “tele-health” OR telemedicine OR “tele-medicine” OR telepractice OR “tele-practice” OR telecare OR “tele-care” OR “tele-monitoring” OR telemonitoring) AND (qualitative OR “focus group discussion” OR “grounded theory” OR ethnography OR phenomenology OR triangulation OR “mixed-methods” OR interview*) AND (rehabilitation OR “occupational therap*” OR “physical therap*” OR “physiotherap*” OR “speech therap*” OR “speech language patholog*”).

A manual search was carried out by reviewing the reference list of included studies. Boolean operators, parenthesis, truncation, and wildcards were used whenever applicable. We consider including published articles after 2010 as there is an exponential rise in the use of TR [23,24]. The search was limited to the article in English and peer-reviewed journals. From there, relevant articles were chosen, and eligibility was determined via a screening procedure.

To ensure that the review integrates the latest evidence in the rapidly developing area, the search was later revised up to 21 February 2026, using the same databases, search terms, and eligibility criteria to ensure methodological consistency.

### 2.4. Study Selection and Screening

PRISMA 2020 was used to guide systematic search, screening, and reporting processes, while qualitative synthesis followed an interpretive thematic meta-synthesis approach [25]. Two authors, Yughdtheswari Muniandy (YM) and Mohd Hibatullah Romli (MHR), independently screened all records. The first authors screened for the study title according to the predetermined selection criteria. Then, two authors independently screened for all the abstracts. If the abstract is not detailed, then the author reads the full text. Following with abstract, screening for full text was carried out to identify the potential article. The final stage involves comparing full-text articles among both the author and any disagreement between the author was discussed until consensus was achieved.

### 2.5. Quality Assessment of Included Studies

Quality assessment is essential to allow for evaluation of the studies and to determine the quality and integrity of the data presented, to eliminate studies whose results are not robust enough. In current study we have assessed the methodological quality of the included studies using the Critical Appraisal Skills Programme (CASP) [26]. CASP has been proven to be a good measure of transparency and reporting standards, and it is one of the most widely used instruments recommended by Cochrane collaboration in appraising qualitative studies [27,28]. CASP is a ten-item checklist with each item assessed using a three-point scale (0 = criterion not met; 1 = criterion partially met; 2 = criterion totally met). There is no overall score, and each interpretation is examined individually. This checklist consists of a set of items that assist the reviewer in determining the study’s rigor, credibility, and relevance [29]. Two authors have independently assessed the methodological quality of the studies, and disagreements between the review author were resolved by discussion, with the involvement of a third review author when necessary.

### 2.6. Extracting and Presenting the Formal Data

We provide a narrative synthesis of the findings from the included studies. Comprehensive data extraction was performed. A data extraction form was used for documenting the extracted information. Key characteristics of the included studies are extracted into a matrix table following establish guidelines for matrix methods in review studies [30]. The extracted information includes information on author, year, the objective of the study, characteristics of participants (patients/caregivers), type of qualitative framework, type of TR and protocol, country, and summary findings of the study.

### 2.7. Data Analysis

The analysis involves carefully reading and rereading each study. One researcher extracted the formal characteristics of the studies, while data extraction and analysis were independently performed by the researcher and compared, respectively. The themes and subthemes from each publication were retrieved during data extraction, and a thematic framework for the full data set was constructed. This required thorough reading of all the included articles, identifying the key themes described in each, and then constructing a thematic framework that incorporated all recognized topics.

### 2.8. Data Synthesis

The data synthesis developed interpretation from the original studies that considered patients’ perceptions reported in the original studies (first-order construct) and then allows the interpretation of patients’ understanding made by the researcher (second-order constructs). This is then followed by the synthesis of both first and second-order constructs into the development of the new interpretations (third-order constructs) that go beyond those offered in individual primary studies and offer reinterpretation based on primary research.

The results of the included studies were analyzed to extract the first-order constructs with a primary focus on the experiences reported by the participants with verbatim quotations where available. These constructs were then coded inductively and then clustered based on conceptual similarity to form second-order constructs (descriptive themes). Through dynamic comparison between studies, correlations between second-level constructs were evaluated, generating third-level constructs (analytic themes) which represented common patterns between contexts.

We summarized the themes and subthemes from each article and condensed them into the overarching themes, as well as how each publication’s authors expressed and interpreted common understandings. The richness of the data and the depth of the explanatory analysis were judged more essential than the frequency with which themes were reported.

### 2.9. Confidence in Review Findings (GRADE-CERQual)

Confidence in each synthesized review finding was assessed using the GRADE-CERQual approach. This framework evaluates confidence in qualitative evidence synthesis findings based on four components: methodological limitations, coherence, adequacy of data, and relevance of the contributing studies. Based on these assessments, each finding was assigned an overall level of confidence (high, moderate, low, or very low).

## 3. Results

### 3.1. Review Identification and Selection

**Figure 1:**
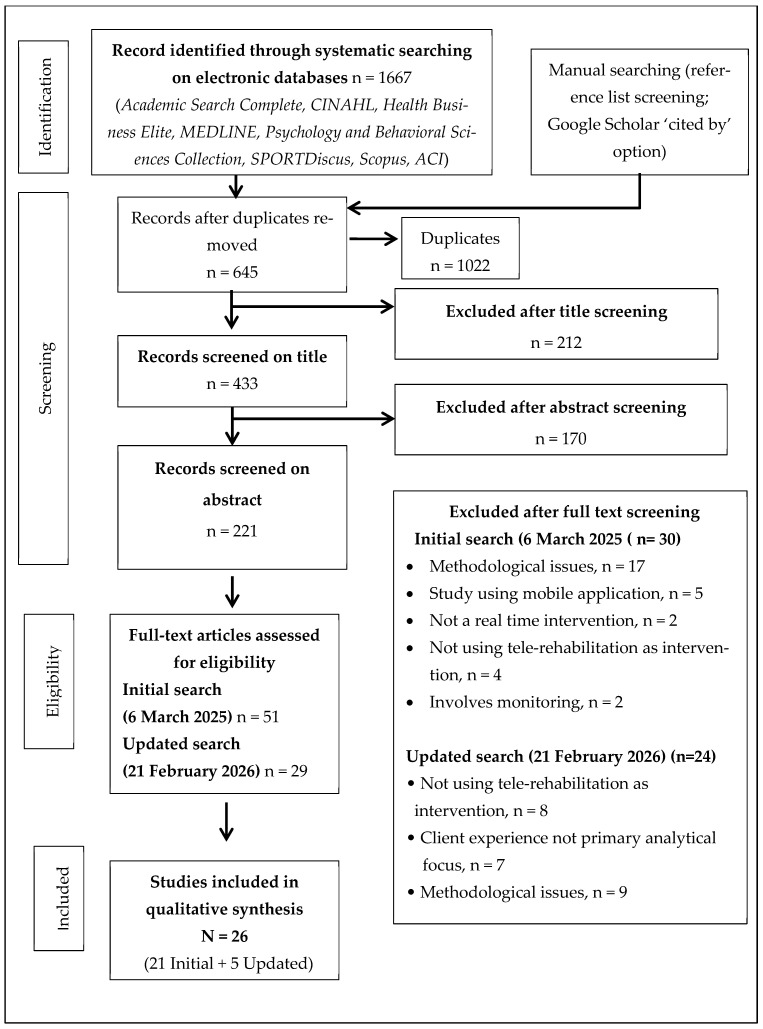
Prisma flowchart for study selection.

The process of identifying and selecting pertinent records for this review was executed through a rigorous and systematic search across various electronic databases. The initial search conducted on 6 March 2025 resulted in a cumulative total of 1667 records. Subsequent to the elimination of duplicates, 645 distinct records were deemed available for the screening process, leading to the evaluation of 51 articles in full text. Among these evaluated articles, 30 were ultimately excluded (see Figure 1). The analysis of the search indicated that while all identified articles conformed to the utilized keywords, the majority failed to satisfy the predetermined inclusion criteria. Consequently, 21 articles were included in initial synthesis. Without the involvement of an unbiased adjudicator, the two authors (YM and MHR) successfully reached a consensus. An updated search was then conducted on 21 February 2026 using the same search strategy and eligibility criteria and 29 full texts articles were assessed to evaluate eligibility and among these 5 studies met the inclusion criteria and were included into the end synthesis. The meta-synthesis ended up including 26 studies in total. The excluded articles, along with reasons for exclusion, have been documented in Appendix A (Table A1 and Table A2).

From our comprehensive literature review, it was determined that TR has been predominantly employed in the fields of neurology, cardiology, speech therapy, and physiotherapy rehabilitation. The duration of the TR interventions varied between 4 to 24 weeks reflecting variability in program intensity and follow-up. Among the collection of articles, the focus on the client’s experience and perspective was oriented towards rehabilitation rather than mere monitoring. The characteristics of the included studies, including the study objectives, participant characteristics, type of qualitative framework, and telerehabilitation interventions, are summarized in Table 1.

### 3.2. Method Quality

Two independent researchers rated the technique quality using the Critical Appraisal Skills Programme instrument, for each included publication. The studies reviewed dis-played a range of quality scores from 14 to 20, with study by Kairy and colleagues achieving the highest score of 20, indicating strong methodology [47]. Most studies scored 80% or above, demonstrating adherence to appraisal criteria. Key findings included clarity of research aims, appropriate methodologies, and effective data collection procedures, with most studies scoring well in these areas. However, some studies fell short in recruitment strategies and detailed ethical considerations. Overall, the assessment using the CASP framework revealed satisfactory methodological quality across included studies, though improvements are needed in specific areas for future research. No research was eliminated due to severe faults in quality. The summary of quality assessment is reported in Table 2.

### 3.3. Thematic Synthesis Findings

After thoroughly reviewing all the articles and identifying the key themes, it was decided that the thematic framework should be based on the client’s perception and experience of using TR. Four key themes emerged: (i) Enhanced Accessibility and reduced Burden; (ii) Perceived Efficiency and Continuity of Care (iii) Therapeutic Relationship and Social Presence (iv) Digital and Contextual Challenges (Table 3).

These themes were developed over an interpretive process starting with the participant-reported experiences (first-order constructs), then developing into descriptive categories (second-order constructs), and finally, analytical themes (third-order constructs). As an example, participant descriptions of long travel distances or time saved by not attending clinical appointments were first coded as reduced travel burden. Subsequently, these codes were clustered into the construct of reduced travel burden and contributed to overarching theme of Enhanced Accessibility and Reduced Burden.

The interventions varied significantly, ranging from home-based exercise programs and individualized therapy sessions to parent coaching and social skills training. The re-ported benefits included improved accessibility, enhanced motivation, and continuity of care. However, challenges were also noted, including technology barriers, lack of hands-on supervision, and privacy issues.

The full thematic analysis matrix, including verbatim quotations, is provided in Appendix B (Table A3).

**Theme 1: Enhanced Accessibility and Reduced Burden**

Participants in the study reported notable benefits from reduced travel requirements for therapy sessions, leading to decreased physical and logistical burdens. This reduction in commuting stress enhances the therapeutic experience, supported by multiple studies [38,39,42,43,47,50]. One participant mentioned, “Travel in country areas is just too hard and having telehealth in the home makes it so easy to do. I can’t do a 70 km round trip” [38]. This aligns with other findings suggesting that telerehabilitation makes healthcare more accessible and significantly reduces the physical effort involved in attending therapy. Additionally, participants experienced notable cost and time savings, allowing for better allocation of resources and improved quality of life [36,38,39,43,50,52,54]. One participant shared, “It takes less out of our day. We have other children and we both work, and we’re pretty busy so to be able to say ‘we’re off to speech therapy now’ and it’s just one hour out of your day rather than three hours, was great” [34]. Reduced transport costs were highlighted, particularly by individuals with mobility challenges or those living in rural areas where travel costs can be high [43]. Flexible scheduling options have improved session adherence by accommodating work and family commitments, as evidenced by various research findings [32,39,49,54]. “Personally, I could never attend in-person sessions because of overlaps with worktime, and so I accepted the moment they told me about the telerehab. It was compatible with my job and unfolded nicely” [32]. Moreover, the ability to schedule sessions around personal commitments allowed individuals to maintain consistency in their rehabilitation. For those individuals in rural or underserved areas, TR has enhanced access that would otherwise have been difficult to obtain due to geographical barriers. One participant noted, “ Accessibility for me. Being able to do it in the morning pretty much from home in the space that I’ve set aside. The benefit is that it’s less travel” [36]. TR was seen as particularly beneficial for individuals with mobility limitations, as it removed the need for long travel distances and provided greater accessibility.

**Theme 2: Perceived Efficiency and Continuity of Care**

The findings of this theme reveal a comprehensive exploration of user perceptions regarding TR services, revealing a complex interplay of advantages and limitations. The subtheme of perceived efficiency reveals mixed opinions on TR’s effectiveness compared to traditional consultations. A significant number of users express appreciation for the convenience and accessibility that TR offers. However, this positive sentiment is tempered by apprehensions surrounding potential drawbacks, including the diminished personal interaction and the risk of miscommunication that can arise in a virtual environment. For instance, one participant stated, “I’m fairly certain that at least twice, on two occasions certainly if he would have come, it would have been a plus” [47]. This sentiment highlights the limitations of TR for physical therapy requiring hands-on manipulation. The autonomy and control highlight a transformative shift in patient engagement as TR empowers individuals to take an active role in managing their treatment plans. Patients frequently report an enhanced sense of self-management when utilizing TR services [40,42]. This newfound autonomy enables them to make informed decisions regarding their healthcare, fostering a sense of ownership and accountability for their health outcomes. Continuity of care is crucial, particularly for chronic condition management, as TR ensures ongoing support and connection with healthcare providers, enriching patient experience and health results. One participant shared, “I feel I’m not being left alone throughout both the social upheaval [in late 2019] and now the pandemic. I have had my therapy uninterruptedly, although virtual, personally I haven’t felt abandoned.” [32]. Additionally, TR facilitates the integration of therapeutic practices into daily routines, helping patients overcome motivational barriers and adhere to treatment plans. This integration makes therapy less intimidating and more manageable, resulting in higher adherence rates and satisfaction with care. However, some participants noted motivation challenges when doing therapy from home. One participant admitted, “…doing it from home isn’t very motivating… It’s a lot harder to concentrate and a lot harder to… get into it” [44]. Despite these challenges, many still valued the flexibility TR provided. A participant mentioned, “If asked ‘would you like to try again,’ I would even though, I mightn’t be fit enough to complete all the exercises, but I still would because it was enjoyable” [51].

**Theme 3: Therapeutic Relationship and Social Presence**

In the exploration of therapeutic practices, the concept of the therapeutic relationship and social presence emerges as a crucial theme. It encompasses the dynamics between the therapist and the client, highlighting the importance of connection, trust, and engagement in the healing process. Participants shared that they felt a strong quality in their therapeutic alliance, emphasizing how crucial trust and emotional backing were in their relationships with therapists. One participant shared, “…we discussed fishing, we discussed hunting, (…) we discussed skiing, um, all kinds of topics, while I was doing my exercises, we had conversations about everything and always found something to talk about. I think she understood my whole life (laughter)” [47]. However, some participants shared their initial doubts about the virtual format, with one saying, “Some stranger appeared on Zoom, and I thought, ugh, I wouldn’t trust them at all” [37]. Even with these worries, many found the teletherapy sessions useful. The quality of communication was a crucial aspect of the therapeutic relationship. Participants noted that clear explanations and effective feedback were essential for successful teletherapy sessions. A participant explained, “…because instead of just showing, you have to explain over Telehealth, and that the explanations sometimes take up a lot more time when it’s difficult to explain or, like, to point on your back where it is. It’s easier to show in-person, which helps to kinda save time…” [44]. This highlights the challenges therapists face in ensuring clarity during virtual sessions. Another participant remarked, “The physiotherapist in the videos demonstrated the exercises slowly and explained things easily. I was really surprised how the residents were able to follow everything without any help” illustrating the effectiveness of well-structured communication [38]. Emotional connection and trust were found to develop over time, with participants recognizing the importance of rapport in their therapeutic relationships. One participant shared, “It was good, just the hug was missing! The hug twice a week” emphasizing the longing for physical connection [34]. Another participant reflected on their long-standing relationship with their doctor, stating, “I’ve known my doctor for over ten years so it’s pretty good rapport, easy prescriptions” [39]. This demonstrates how established relationships can foster trust and enhance therapeutic outcomes. The ability to involve family members in TR sessions was a major benefit, particularly for those who required caregiver support. One participant shared, “I appreciate it when my husband keeps pushing and motivating me to keep training and following my goals for my rehabilitation” [40]. This reflects the benefits of TR in terms of engaging caregivers in the rehabilitation process.

**Theme 4: Digital and Contextual Challenges**

This theme captures the different digital and situational obstacles encountered by participants during TR sessions. Participants reported several technological barriers and enablers that impacted their TR experiences. Issues such as poor internet connectivity were common, with one participant stating, “a few rough days there that third week where the connection wasn’t fantastic, it kept pausing” [54]. Others noted specific glitches, such as, “Once my computer kept blacking out and I had to keep coming, leaving… but I could just trot over here and do my button here [indicates Zoom© icon] and it would come back on” … But that was the only glitch [55]. While some participants enjoyed a seamless experience due to reliable internet, as one remarked, “For me, it was good because my internet is good. So, there was no problem”. The use of technology for better engagement was also mentioned, with a participant stating, “Zoom worked well when we connected the iPad to the TV, we were able to turn the volume of the TV up so the residents could hear better. It also gives a bigger picture as well, so they can see the physio better” [38]. Concerns about privacy were also prevalent, as one participant ex-pressed, “I’m concerned about privacy, of course right. Especially knowing that even Zoom had its own privacy and confidential issues” [39]. Contextual challenges primarily revolved around the difficulties in assessing or correcting physical movements during TR sessions. One participant articulated this concern, “In terms of physiotherapy, not having someone walking beside you and making corrections as you go along […] it was less, I felt like there was less potential for feedback in terms of physiotherapy” [52]. The inability to provide hands-on assistance was highlighted as a significant drawback, with one participant stating, “the disadvantage I see is that physical assessment isn’t fully done, the one that involves touch, palpation and actually see in detail if I’m doing my exercise correctly” [32]. Digital literacy emerged as a critical factor influencing the TR experience. One participant remarked, “Obviously they would need to be confident in the software themselves, to be able to advise people having difficulties because sometimes these things do drop out and they can be a real impediment to relationship if it’s just frustrating” [50]. Concerns about technological proficiency were evident, with one participant stating, “I’m technophobic—my daughter did a lot of it for me at the beginning” [55]. Another participant reflected on the complexities faced by elderly users, saying, “For me, everything was complex […], an elderly person who is not accustomed to technology […], I think it would be challenging” [52]. This highlights the need for digital literacy support for patients to maximize the effectiveness of TR. Home environment constraints also played a significant role in shaping the TR experience. Participants reported distractions and limitations in physical space and equipment. One participant shared, “I think it’s just distraction. With my husband in the office and then with […]” [49]. Another noted, “I’m kind of limited with the equipment that I have at home, it is somewhat limiting in what I can do with the exercise program that I’ve got” [36]. The availability of adequate equipment was also a concern, as indicated by one participant who said, “The equipment we usually use, either we don’t find them or are very costly” [32].

The confidence in each synthesized finding was assessed using the GRADE-CERQual framework and the results are presented in Table 4. Individual studies contributed to more than one theme, reflecting the multidimensional nature of client experiences with telerehabilitation.

## 4. Discussion

This meta-synthesis illustrates that client experiences of telerehabilitation (TR) are conditioned by a multifaceted interaction of the accessibility, relational dynamic and contextual constraints. TR is neither beneficial nor harmful; its appropriateness depends on the compatibility between patient-clinical needs and conditions of implementation. It is imperative to understand these experiential dimensions to inform the refinement and sustainable integration of telerehabilitation.

Accessibility has become one of the key themes. Less travel load, time conservation and scheduling flexibility were commonly documented [57,58]. According to the access framework offered by Penchansky and Thomas (1981), TR enhances geographic accessibility by removing the necessity to travel, contributes to increasing the structural match between services and user routines [59]. Affordability was mainly connected with a decrease in transport cost, and work loss instead of actual healthcare costs. Notably, perceived savings cannot be referred to as system-level efficiency. According to Sanchez-Ferre et al. (2025), formal cost-effectiveness of TR programs is needed as the economic benefits of these programs are always context-specific [60]. Engagement to TR was dependent on stable internet, digital competency and home suitability [61]. Transportation expenses are shifted to the purchase of technologies and connectivity in low-income families [57,58,62]. In line with digital health equity literature, implementation has the potential to increase inequity in cases where structural determinants are not addressed [62,63]. Basic infrastructures, such as lack of internet connection, electricity supply, and secure platforms, are core hindrances to sustainability in low- and middle-income nations [58,62,64]. Contrarily, higher resource settings emphasized organization issues [61,63,65,66]. Although TR eliminates geographic obstacles, its successful implementation depends on infrastructure and resources capacity.

Perceived efficiency and continuity of care reflect is evident by reduced logistical disruption and integration into everyday activities [62]. Efficiency is described in terms of minimized level of personal burden and flexibility in scheduling, as opposed to enhanced resource utilization in the system level. Participants felt higher sense of ownership of their rehabilitation process when exercises could be performed at home environment [56]. Nonetheless, autonomy does not exist within an independent context of self-care; instead, it exists in the context of a structured therapeutic relationship. Continuous monitoring and feedback are crucial in maintaining engagement [65,67]. Continuity depends on the reliability of the technology, while interruptions can interfere with the therapeutic progress [57,58]. Participants expressed lack of motivation in absence of an in-person therapist, indicating that self-management ability are dependent on the situation and not the individual traits [56,68,69]. Telerehabilitation may be more accurately conceptualized as facilitating supported self-management.

Therapeutic relationships represent an ambiguous and arguably paradoxical field. Some participants experience lower rapport, less emotional urgency, and relational distance, while others noticed sustained trust and meaningful connection in virtual communication [56,66]. The therapeutic relationship in telerehabilitation does not follow a standard curve but varies according to the situation. The success of the transition was more effective when pre-existing relationships were established in comparison to newly initiated ones [67]. The quality of communication, such as clarity of instruction, organized feedback, and demonstration, improves the strength of alliances [66]. TR also facilitates greater caregiver engagement compared with the traditional clinician–patient dyad [70]. In general, establishing therapeutic relationships in virtual settings requires using adaptive relational strategies that correspond to the patient expectations and clinical situation.

Technology became an inhibitor and facilitator. The instability of connectivity and complexity in the platform interfered with engagement [65,66]. Meanwhile, digital features like screen sharing, recording and a connection with monitoring devices provided more engagement and flexibility [68]. Thus, suggesting that effective telerehabilitation is dependent on contextual and organizational factors as opposed to technological characteristics alone [63]. Organizational determinants including staffing shortage, limited training, and lack of clinical protocols hindered adoption and large-scale implementation [64]. Digital preparedness is thus not limited to connectivity, it also implies workforce proficiency and institutional encouragement.

The complexity is further described by age differences and clinical conditions. Digital literacy issues and technology hesitancy are more frequently reported by older people [61,71]. Stroke survivors describe cognitive load associated with navigating digital interfaces [14]. However, these obstacles can be reduced with systematic training and technical assistance [71]. Digital capability is not strongly predicted by chronological age and combination of socioeconomic status, education, and previous technology exposure influences digital literacy [66]. The determinants of feasibility are also contingent. Musculoskeletal rehabilitation was constrained by inability to perform hands-on assessment [66]. Conversely, cardiac and respiratory rehabilitation, emphasizing exercises prescription adapted more readily to remote delivery [65,69]. Neurological populations benefited from continuity but experienced communication difficulties in cases of cognitive or language impairment [56]. These variances reinforce the implementation of condition-sensitive hybrid models as opposed to standard deployment.

### 4.1. Implications and Future Directions

Collectively, these findings indicate that telerehabilitation should be implemented as a context-sensitive modality rather than a universal substitute for in-person care. Clinical stratification based on functional capacity, digital literacy, social support, and condition-specific requirements are essential. Hybrid models integrating periodic in-person evaluation may optimize safety and effectiveness across heterogeneous populations. Intentional relational strategies, technical support systems, and workforce training are necessary to sustain quality.

Future research should compare virtual, in-person, and hybrid rehabilitation models across different conditions to determine which approach is most effective. Longitudinal studies are needed to understand more efficiently how therapeutic relationships are formed and preserved in the digital environment. The role of organizational structures and system level variables in promoting sustainable implementation should also be researched. Lastly, the assessment of the long-term outcomes and burden of caregiving is also essential.

### 4.2. Strengths and Limitations

This meta-synthesis has several strengths. Methodological transparency was enhanced through a systematic search in numerous databases and quality appraisal through CASP. This inclusion of 26 qualitative studies covering a wide range of population, clinical conditions, and geographic settings offers a wide-range base of evidence. The synthesis goes beyond description to produce a higher order of analytical provision by incorporating findings through theoretical interpretation and investigating inconsistencies among studies. Focusing on client views contributes more towards its applicability with patient-centered implementation.

Several limitations should be acknowledged. Most included studies were conducted in high-income countries, limiting transferability to low- and middle-income contexts where infrastructure and resource constraints differ substantially. Additionally, many studies were undertaken during or shortly after the COVID-19 pandemic, when tele-rehabilitation was rapidly deployed; experiences may evolve as implementation becomes more structured and sustainable. As with all qualitative syntheses, interpretive analysis may yield different insights under alternative theoretical lenses. Finally, heterogeneity across interventions and populations limits the ability to draw condition-specific or modality-specific conclusions.

## 5. Conclusions

Telerehabilitation reconfigures rehabilitation delivery rather than simply adding technology. Its success depends on alignment between patient capability, clinical needs, and organizational support. With adequate infrastructure and relational adaptation, it can improve access and continuity. However, if digital and contextual barriers are not addressed, telerehabilitation may reinforce existing inequities.

## Figures and Tables

**Table 1:** Study Characteristics of studies on client’s experience using telerehabilitation (TR) is reviewed.

Author(s), Year Country	Study Aim	Study Population	Rehabilitation Area	Intervention	Reported Benefits (Positive Experiences)	Reported Disadvantages (Negative Experiences)
Aarabi MA et al., 2025 [31] Iran	To examine the challenges of telerehabilitation	Children with physical disabilities	Occupational therapy	Telerehabilitation program (Detail not specified)	Continuous access to rehabilitation; increased accessibility, reduced time burden	Lack of a developed program; Unorganized internet infrastructure
Aliaga-Castillo et al., 2022 [32] Chile	To evaluate the usefulness, safety, effectiveness, and limitations of a TR program	Adults with severe haemophilia	Physiotherapy	Home-based strength and balance training with education	Improved physical and mental well-being continuity of care; cost and time savings	Lack of hands-on supervision; short session duration; limited equipment
Angell et al., 2024 [33] USA	To examine experiences of telehealth-delivered pediatric therapy	Autistic children and parents	Occupational therapy	Parent coaching and co-regulation strategies	Improved parent engagement; family- centered care	Parental stress; difficulty with hands-on intervention
Barboza et al., 2024 [34] Brazil	To explore experiences of TR in Parkinson’s disease	Adults with Parkinson’s disease	Physiotherapy	Motor, stretching, and cognitive exercises	High satisfaction; empowerment; emotional well-being	Poor internet connectivity; lack of physical contact
Brehon et al., 2026 [35] Canada	To understand the perceptions and lived experiences regarding telerehabilitation	Adults with posttraumatic stress injuries	Multidisciplinary Rehabilitation	Interdisciplinary rehabilitation programs	Increased accessibility, reduced time burden; ability to engage in vulnerable conversations	Difficulty observing subtle cues and fostering therapeutic alliance
Brown et al., 2023 [36] Australia	To explore experiences of telehealth exercise physiology	Adult clients	Exercise Physiology	Individualized exercise programs	Improved access; flexibility; cost effectiveness	Technological disruptions; rapport challenges
Daly et al., 2022 [37] Ireland	To explore service users’ experiences of telehealth	Adults in mental health services	Occupational Therapy	Functional activity-based therapy	Improved accessibility; continuity of care	Privacy concerns; reduced human connection
Dawson et al., 2024 [38] Australia	To describe experiences and acceptability of telephysiotherapy	Older adults	Physiotherapy	Balance and strength exercises	Improved adherence; motivation	Technology hesitancy; sensory impairments
Dean et al., 2024 [39] Canada	To explore experiences of teleSCI services	Adults with spinal cord injury	Multidisciplinary Rehabilitation	Remote medical and therapy services	Improved convenience; reduced travel	Reduced human connection; technology barriers
Dinesen et al., 2019 [40] Denmark	To explore experiences of cardiac TR	Adults with cardiac disease	Cardiac Rehabilitation	Exercise and education program	Enhanced autonomy; continuity of care	Motivational challenges; digital literacy barriers
Erden Guner et al., 2025 [41] Turkey	To explore perceptions and experiences regarding telerehabilitation	Children with Duchenne Muscular Dystrophy (DMD	Physiotherapy	Exercises	Improved access to exercise; enhanced psychosocial well-being; increased motivation.	Internet interruptions and connectivity issues; hesitation due to camera visibility
Eriksson et al., 2011 [42] Sweden	To explore patient experiences of home-based TR	Post shoulder replacement adults	Physiotherapy	Video-based physiotherapy	Increased safety and empowerment	Connectivity issues; communication challenges
Gaboury et al., 2022 [43] Canada	To describe acceptability of stroke TR	Adults post-stroke	Multidisciplinary Rehabilitation	Neuromuscular facilitation	High satisfaction; functional gains	Low technology familiarity
Hudon et al., 2024 [44] Canada	To explore injured workers’ experiences of TR	Adults with work-related injuries	Multidisciplinary Rehabilitation	Education and exercise	Continuity of care; flexibility	Perceived inferiority to in-person care
Jansen-Kosterink et al., 2019 [45] Netherlands	To explore perceptions of telemedicine in rehabilitation	Adults with chronic disease	Multidisciplinary Rehabilitation	Home exercise with telemonitoring	Convenience; personalized feedback	Motivation and privacy concerns
Johnsson et al., 2019 [46] Australia	To evaluate feasibility of tele-therapy for autism	Autistic children and families	Multidisciplinary Rehabilitation	Behavioral support and social skills training	Improved access to specialists	Scheduling and rapport challenges
Kairy et al. 2013 [47] Canada	To explore patients’ perceptions regarding in-home TR services.	Adults following total knee arthroplasty	Physiotherapy	In-home TR exercise program	Improved access; strong therapeutic relationship	Perceived need for some in-person visits for comprehensive assessment; technical issues
Kringle et al., 2023 [48] USA	To explore stakeholder experiences in group TR	Adults post-stroke	Occupational Therapy	Social learning and guided problem- solving	Peer learning; engagement, Improved access and flexibility	Technology disruptions
Kwok et al., 2022 [49] Canada	To explore tele-practice for communication disorders	Preschool children	Speech and Language Therapy	Speech, language, and communication intervention program	Improved parent collaboration; Improved access and flexibility	Assessment limitations; distractions
Lee et al., 2022 [50] Australia	To explore telehealth physiotherapy for bronchiectasis	Adults with bronchiectasis	Physiotherapy	Airway clearance therapy	High satisfaction; Continuity of care	Lack of hands-on assessment; Technology disruptions
O’Shea et al., 2022 [51] Ireland	To explore virtual pulmonary rehabilitation	Adults with Pulmonary fibrosis	Physiotherapy	Pulmonary rehab exercises	Improved motivation; and high satisfaction	Lack of social connection and technical issues.
Ouédraogo et al., 2024 [52] Canada	To explore acceptability of stroke TR	Adults post-stroke	Multidisciplinary Rehabilitation	Home-based TR	Functional improvement and increase accessibility	Internet instability and lack of equipment
Reis et al., 2025 [53] Portugal	To understand how older people perceive the implementation of telerehabilitation	Older adults with Chronic Obstructive Pulmonary Disease	Respiratory rehabilitation	Exercises and education	Overcomes geographical and physical barriers; Allows continuity of care	Lack of familiarity with technology; data privacy and security concerns
Thomas et al., 2018 [54] Australia	To explore telehealth experiences	Children with Childhood Apraxia of Speech	Speech and Language Therapy	Motor-learning-based speech therapy	Convenience and time-efficiency	Parent discomfort and less cooperative
Wiley et al., 2024 [55] Canada	To explore TR after stroke	Adults post-stroke	Physiotherapy	Exercise and self-management	Improved mobility and Continuity of care	Lack of physical touch
Wong AK et al., 2025 [56] Hong Kong	To explore the experiences of survivors of stroke with teleconsultation program	Adults post-stroke	Nurses	Education and counseling	Reduced logistical burdens and enhanced access to care; Increased awareness and motivation	Digital and usability barriers; inability to perform physical assessments

**Table 2:** Quality rating of each study using Critical Appraisal Skills Programme (CASP).

Studies	Evaluation Criteria (Score 0 = Not Met; 1 = Partially Met; 2 = Totally Met)										Total (/20) Percentage (100%)
	Item 1	Item 2	Item 3	Item 4	Item 5	Item 6	Item 7	Item 8	Item 9	Item 10	
Aarabi MA et al., 2025 [31]	2	2	1	1	2	0	2	1	2	1	14 (70%)
Aliaga-Castillo et al., 2022 [32]	2	2	1	2	2	1	1	1	2	2	16 (80%)
Angell et al., 2024 [33]	2	2	1	2	2	1	1	1	2	2	16 (80%)
Barboza et al., 2024 [34]	2	2	2	2	2	1	1	1	2	2	17 (85%)
Brehon et al., 2026 [35]	2	2	2	1	2	1	2	2	2	2	18 (90%)
Brown et al., 2023 [36]	2	2	2	2	2	1	1	1	2	2	17 (85%)
Daly et al., 2022 [37]	2	2	2	1	2	1	1	1	2	2	16(80%)
Dawson et al., 2024 [38]	2	2	2	2	2	1	1	1	2	2	17 (85%)
Dean et al., 2024 [39]	2	2	1	2	2	1	1	1	2	2	16 (80%)
Dinesen et al., 2019 [40]	2	2	2	2	2	1	2	2	2	1	18 (90%)
Erden Guner et al., 2025 [41]	2	2	2	2	2	1	2	2	2	2	19 (95%)
Eriksson et al., 2011 [42]	2	2	2	1	2	1	1	1	2	2	16 (80%)
Gaboury et al., 2022 [43]	2	2	2	2	2	1	1	1	2	2	17 (85%)
Hudon et al., 2024 [44]	2	2	2	2	2	1	1	1	2	2	17 (85%)
Jansen-Kosterink et al., 2019 [45]	2	2	2	1	1	1	2	1	2	2	16 (80%)
Johnsson et al., 2019 [46]	2	2	2	1	2	1	2	2	2	1	17 (85%)
Kairy et al. 2013 [47]	2	2	2	2	2	2	2	2	2	2	20 (100%)
Kringle et al., 2023 [48]	2	2	2	2	2	1	1	1	2	2	17 (85%)
Kwok et al., 2022 [49]	2	1	2	2	2	1	1	1	2	2	16 (80%)
Lee et al., 2022 [50]	2	2	1	2	2	1	1	1	2	2	16 (80%)
O’Shea et al., 2022 [51]	2	1	2	2	2	1	1	1	2	2	16 (80%)
Ouédraogo et al., 2024 [52]	2	2	1	2	2	1	1	2	2	2	17 (85%)
Reis et al., 2025 [53]	2	2	2	1	2	1	2	2	2	2	18 (90%)
Thomas et al., 2018 [54]	2	2	2	2	2	1	1	2	2	1	17 (85%)
Wiley et al., 2024 [55]	2	2	1	2	2	1	1	1	2	2	16 (80%)
Wong AK et al., 2025 [56]	2	2	2	1	2	1	2	2	2	2	18 (90%)

Item 1: Was there a clear statement of the aims of the research; Item 2: Is a qualitative methodology appropriate; Item 3: Was the research design appropriate to address the aims of the research; Item 4: Was the recruitment strategy appropriate to the aims of the research; Item 5: Was the data collected in a way that addressed the research issue; Item 6: Has the relationship between researcher and participants been adequately considered; Item 7: Have ethical issues been taken into consideration; Item 8: Was the data analysis sufficiently rigorous; Item 9: Is there a clear statement of findings; Item 10: How valuable is the research?

**Table 3:** Integrated Thematic Synthesis of Client Experiences of Telerehabilitation.

Third-Order Constructs (Analytical Themes)	Second-Order Constructs (Descriptive-Themes)	First-Order Constructs (Participant-Reported Experiences)	Articles
Theme 1: Enhanced Accessibility and Reduced Burden	Reduced travel burden	Absence of travel, reduced physical and logistical effort to attend therapy	Eriksson et al., 2011 [42]; Kairy et al., 2013 [47]; Gaboury et al., 2022 [43]; Lee et al., 2022 [50]; Wiley et al., 2024 [55]; Dean et al., 2024 [39]; Dawson et al., 2024 [38]; Erden Guner et al., 2025 [41]; Wong AK et al., 2025 [56]; Reis et al., 2025 [53]; Aarabi MA et al., 2025 [31]
	Cost and time savings	Reduced transport costs, time saved from commuting	Thomas et al., 2018 [54]; Gaboury et al., 2022 [43]; Brown et al., 2023 [36]; Lee et al., 2022 [50]; Ouédraogo et al., 2024 [52]; Dawson et al., 2024 [38]; Dean et al., 2024 [39] Wong AK et al., 2025 [56]; Reis et al., 2025 [53]; Aarabi MA et al., 2025 [31]
	Scheduling flexibility and improved session adherence	Easier scheduling, better compatibility with work and family responsibilities, and reduced disruptions to daily routines.	Thomas et al., 2018 [54]; Aliaga-Castillo et al., 2022 [32]; Kwok et al., 2022 [49]; Dean et al., 2024 [39]; Erden Guner et al., 2025 [41]; Reis et al., 2025 [53]
	Rural and remote access	Improved access for rural, remote, or mobility-limited populations	Dinesen et al., 2016 [40]; Lee et al., 2022 [50]; Ouédraogo et al., 2024 [52]; Wiley et al., 2024 [55]; Brown et al., 2023 [36]; Wong AK et al., 2025 [56]; Reis et al., 2025 [53]; Aarabi MA et al., 2025 [31]; Brehon et al., 2026 [35]
Theme 2: Perceived Efficiency and Continuity of Care	Perceived efficiency	mixed perceptions of TR efficiency, highlighting both the convenience and limitations compared to in-person sessions.	Thomas et al., 2018 [54]; Johnsson et al., 2019 [46]; Gaboury et al., 2022 [43]; Lee et al., 2022 [50]; O’Shea et al., 2022 [51]; Dawson et al., 2024 [38]; Erden Guner et al., 2025 [41]; Wong AK et al., 2025 [56]; Reis et al., 2025 [53]; Aarabi MA et al., 2025 [31]; Brehon et al., 2026 [35]
	Autonomy and control	Greater control over treatment plans and enhanced self-management	Eriksson et al., 2011 [42]; Dinesen et al., 2016 [40]; Jansen-Kosterink et al., 2019 [45]; Barboza et al., 2024 [34]; Gaboury et al., 2022 [43]; Wong AK et al., 2025 [56]; Reis et al., 2025 [53]
	Continuity of care	Ongoing support through TR, especially during periods when in-person care was unavailable	Eriksson et al., 2011 [42]; Kairy et al., 2013 [47]; Daly et al., 2022 [37]; Gaboury et al., 2022 [43]; O’Shea et al., 2022 [51]; Ouédraogo et al., 2024 [52]; Wiley et al., 2024 [55]; Hudon et al., 2024 [44]; Erden Guner et al., 2025 [41]; Wong AK et al., 2025 [56]; Reis et al., 2025 [53]; Aarabi MA et al., 2025 [31]; Brehon et al., 2026 [35]
	Integration into daily routines; overcoming motivation challenges	Therapy embedded into home and daily life	Thomas et al., 2018 [54]; Barboza et al., 2024 [34]; Aliaga-Castillo et al., 2022 [32]; Gaboury et al., 2022 [43]; Ouédraogo et al., 2024 [52]; O’Shea et al., 2022 [51]; Wiley et al., 2024 [55]; Hudon et al., 2024 [44]; Erden Guner et al., 2025 [41]; Wong AK et al., 2025 [56]
Theme 3: Therapeutic Relationship and Social Presence	Perceived quality of therapeutic alliance	Therapeutic alliance, trust and emotional support	Kairy et al., 2013 [47]; Johnsson et al., 2019 [46]; Barboza et al., 2024 [34]; Daly et al., 2022 [37]; Aliaga-Castillo et al., 2022 [32]; Wiley et al., 2024 [55]; O’Shea et al., 2022 [51]; Reis et al., 2025 [53]
	Communication quality	Clear explanations; improved discussion and feedback	Thomas et al., 2018 [54]; Angell et al., 2024 [33]; Wiley et al., 2024 [55]; Aliaga-Castillo et al., 2022 [32]; Erden Guner et al., 2025 [41]; Wong AK et al., 2025 [56]
	Emotional connection and trust	Development of rapport and confidence over time	Thomas et al., 2018 [54]; Barboza et al., 2024 [34]; Aliaga-Castillo et al., 2022 [32]; Ouédraogo et al., 2022 [52]; Dawson et al., 2024 [38]; Aarabi MA et al., 2025 [31]; Brehon et al., 2026 [35]
	Family/caregiver involvement	Caregivers more involved in sessions conducted at home	Dinesen et al., 2016 [40]; Angell et al., 2024 [33]; Barboza et al., 2024 [34]; O’Shea et al., 2022 [51]; Brehon et al., 2026 [35]; Erden Guner et al., 2025 [41]
Theme 4: Digital and Contextual Challenges (cross-cutting)	Digital challenges	Technological Barriers and Enablers in TR	Jansen-Kosterink et al., 2019 [45]; Barboza et al., 2024 [34]; Kringle et al., 2023 [48]; Wiley et al., 2024 [55]; Erden Guner et al., 2025 [41]; Wong AK et al., 2025 [56]; Reis et al., 2025 [53]; Aarabi MA et al., 2025 [31]; Brehon et al., 2026 [35]
	Contextual challenges	Difficulty assessing or correcting physical movements	Kairy et al., 2013 [47]; Thomas et al., 2018 [54]; Daly et al., 2022 [37]; Aliaga-Castillo et al., 2022 [32]; Brown et al., 2023 [36]; Dean et al., 2024 [39]; Brehon et al., 2026 [35]; Erden Guner et al., 2025 [41]; Wong AK et al., 2025 [56]
	Digital literacy issues	Challenges using technology, especially among some users	Johnsson et al., 2019 [46]; Kringle et al., 2023 [48]; Gaboury et al., 2022 [43]; Lee et al., 2022 [50]; Ouédraogo et al., 2024 [52]; Wiley et al., 2024 [55]; Dean et al., 2024 [39]; Reis et al., 2025 [53]; Aarabi MA et al., 2025 [31]
	Home environment constraints	Distractions; limited space or equipment	Thomas et al., 2018 [54]; Brown et al., 2023 [36]; Kwok et al., 2022 [49]; Wiley et al., 2024 [55]; Erden Guner et al., 2025 [41]; Wong AK et al., 2025 [56]

**Table 4:** Summary of qualitative findings and confidence in the evidence (GRADE-CERQual).

Review Finding	Studies Contributing to the Finding	CERQual Assessment (Methodological Limitations, Coherence, Adequacy, Relevance)	Overall Confidence	Explanation of Confidence Judgement
Enhanced accessibility and reduced burden	Eriksson et al., 2011 [42]; Kairy et al., 2013 [47]; Dinesen et al., 2016 [40]; Thomas et al., 2018 [54]; Gaboury et al., 2022 [43]; Lee et al., 2022 [50]; Brown et al., 2023 [36]; Aliaga-Castillo et al., 2022 [32]; Kwok et al., 2022 [49]; Ouédraogo et al., 2024 [52]; Wiley et al., 2024 [55]; Dean et al., 2024 [39]; Dawson et al., 2024 [38]; Erden Guner et al., 2025 [41]; Wong AK et al., 2025 [56]; Reis et al., 2025 [53]; Aarabi MA et al., 2025 [31]; Brehon et al., 2026 [35]	Minor concerns across domains	High Confidence	Supported by a large number of studies reporting consistent experiences of reduced travel burden, increased convenience, and improved access to rehabilitation services.
Perceived efficiency and continuity of care	Eriksson et al., 2011 [42]; Kairy et al., 2013 [47]; Dinesen et al., 2016 [40]; Thomas et al., 2018 [54]; Johnsson et al., 2019 [46]; Jansen-Kosterink et al., 2019 [45]; Aliaga-Castillo et al., 2022 [32]; Daly et al., 2022 [37]; Gaboury et al., 2022 [43]; Lee et al., 2022 [50]; O’Shea et al., 2022 [51]; Barboza et al., 2024 [34]; Dawson et al., 2024 [38]; Ouédraogo et al., 2024 [52]; Wiley et al., 2024 [55]; Hudon et al., 2024 [44]; Erden Guner et al., 2025 [41]; Wong AK et al., 2025 [56]; Reis et al., 2025 [53]; Aarabi MA et al., 2025 [31]; Brehon et al., 2026 [35].	Minor methodological limitations; moderate concerns regarding coherence; minor concerns regarding adequacy of data and relevance.	Moderate Confidence	Participants reported both perceived efficiency and continuity benefits as well as contextual limitations influencing service delivery.
Therapeutic relationship and social presence	Kairy et al., 2013 [47]; Dinesen et al., 2016 [40]; Thomas et al., 2018 [54]; Johnsson et al., 2019 [46]; Aliaga-Castillo et al., 2022 [32]; Daly et al., 2022 [37]; O’Shea et al., 2022 [51]; Ouédraogo et al., 2024 [52]; Barboza et al., 2024 [34]; Angell et al., 2024 [33]; Wiley et al., 2024 [55]; Dawson et al., 2024 [38]; Erden Guner et al., 2025 [41]; Wong AK et al., 2025 [56]; Reis et al., 2025 [53]; Aarabi MA et al., 2025 [31]; Brehon et al., 2026 [35]	Minor methodological limitations; moderate concerns regarding coherence; minor concerns regarding adequacy of data and relevance.	Moderate Confidence	Findings indicate variability in experiences of therapeutic interaction, with some participants reporting preserved rapport and others perceiving reduced interpersonal connection.
Digital and contextual challenges	Jansen-Kosterink et al., 2019 [45]; Kairy et al., 2013 [47]; Thomas et al., 2018 [54]; Johnsson et al., 2019 [46]; Aliaga-Castillo et al., 2022 [32]; Brown et al., 2023 [36]; Daly et al., 2022 [37]; Gaboury et al., 2022 [43]; Lee et al., 2022 [50]; Kwok et al., 2022 [49]; Kringle et al., 2023 [48]; Barboza et al., 2024 [34]; Ouédraogo et al., 2024 [52]; Wiley et al., 2024 [55]; Dean et al., 2024 [39] Erden Guner et al., 2025 [41]; Wong AK et al., 2025 [56]; Reis et al., 2025 [53]; Aarabi MA et al., 2025 [31]; Brehon et al., 2026 [35]	Minor concerns across domains	High Confidence	Consistent reports of technological barriers, digital literacy challenges, and environmental constraints affecting telerehabilitation implementation.

## Data Availability

No new data were created or analyzed in this study. Data sharing is not applicable to this article.

## Abbreviations

The following abbreviations are used in this manuscript:

TR	Telerehabilitation
CASP	Critical Appraisal Skills Programme (CASP)

## Appendix A

### Appendix A.1

Studies were excluded during full-text screening if they did not meet the inclusion criteria. Reasons for exclusion are summarized below.

**Table A1:** List of excluded studies with reason for exclusion.

Reason for Exclusion	Total Study(s)	Excluded Study(s)
Not a real time intervention	2	Bourke A et al., 2023 [72]; Flores-Sandoval et al., 2024 [73]
Study involves monitoring purpose	2	VanRavenstein et al., 2018 [74]; Shulver et al., 2017 [75]
Not using tele-rehabilitation as intervention	4	Moecke et al., 2024 [76]; Ekegren et al., 2023 [77]; Dennett et al., 2024 [78]; Cruickshank et al., 2024 [79]
Study using mobile web applications and others	5	Tyagi et al., 2018 [80]; Ownsworth et al., 2020 [81]; Knudsen et al., 2019 [82]; Kelly et al., 2024 [83]; Brouns et al., 2018 [84]
Methodological issues	17	Verweel et al., 2024 [85]; Phillips et al., 2022 [86]; Philip et al., 2023 [87]; Parraguez et al., 2023 [88]; Palmcrantz et al., 2017 [89]; O’Brien et al., 2023 [90]; Nalder et al., 2018 [91]; Killingback et al., 2024 [92]; Khan et al., 2022 [93]; Fairweather et al., 2017 [94]; Chen PH et al., 2024 [95]; Chen Y et al., 2020 [96]; Ceprnja D et al., 2023 [97]; Castro MJ et al., 2023 [98]; Candelaria et al., 2022 [99]; Brown J et al., 2022 [100]; Barton CJ et al., 2022 [101]

### Appendix A.2

Studies were excluded during full-text screening for the updated search (21 February 2026) if they did not meet the inclusion criteria. Reasons for exclusion are summarized below.

**Table A2:** List of excluded studies with reason for exclusion (Updated search up to 21 February 2026).

Reason for Exclusion	Total Study(s)	Excluded Study(s)
Not using telerehabilitation as intervention	8	Sextl-Plotz et al., 2025 [102]; Joensen ED et al., 2025 [103]; Breder K et al., 2025 [104]; Pang NQ et al., 2025 [105]; Akgun I et al., 2025 [106]; Zheng X et al., 2026 [107]; Ong T et al., 2025 [108]; Podar MD et al., 2025 [109]
Client experience not primary analytical focus	7	Gamble CJ et al., 2025 [110]; Lemersre P et al., 2025 [111]; Reitzel M et al., 2025 [112]; Salahshoori F et al., 2025 [113]; Stark-Blomeier AL et al., 2025 [114]; Onal B et al., 2025 [115]; McLaughlin KH et al., 2026 [116]
Methodological issues	9	Gregersen S et al., 2025 [117]; Micheluzzi V et al., 2026 [118]; Steinberg et al., 2025 [119]; Ouendi et al., 2025 [120]; Gungor Ketenci et al., 2025 [20]; Yap S et al., 2025 [121]; Okusa S et al., 2025 [122]; Mustafa MM et al., 2025 [123]; Gul H et al., 2025 [124]

## Appendix B

This appendix presents the full thematic analysis matrix derived from the meta-synthesis of included qualitative studies.

**Table A3:** Thematic analysis client’s perception and experience using telerehabilitation.

Third-Order Constructs (Analytical Themes)	Second-Order Constructs (Descriptive-Themes)	First-Order Constructs (Participant-Reported Experiences)	Verbatim Quotes	Articles
Theme 1: Enhanced Accessibility and Reduced Burden	Reduced travel burden	Absence of travel, reduced physical and logistical effort to attend therapy	“Travel in country areas is just too hard and having telehealth in the home makes it so easy to do. I can’t do a 70 km round trip—it is too expensive”.	Dawson et al., 2024 [38]
			“I mean the best part about it for me, I didn’t have to travel to the centre for treatment. I could do it from home, which certainly was very convenient. So that was good.”	Lee et al., 2022 [50]
			“I really like it (telerehabilitation). I found it fantastic…you know, just the fact of not having to travel when we are in pain (…) I adored it…”	Kairy et al. 2013 [47]
	Cost and time savings	Reduced transport costs, time saved from commuting	“It takes less out of our day. We have other children and we both work, and we’re pretty busy so to be able to say, “we’re off to speech therapy now” and it’s just one hour out of your day rather than three hours, was great”.	Thomas et al., 2018 [54]
			“I am probably saving at least an hour. I would spend an hour between travel [and] parking, waiting for a fifteen-minute appointment. Whereas now I just pick up the phone, do the appointment, and go right back to work or right back to my life”	Dean et al., 2024 [39]
			“So, if I can do a lot of it over the telephone then it saves us a lot of time and money”	Dean et al., 2024 [39]
			“Avoided travel and associated costs, as well as better health safety due to avoided contamination”.	Reis et al., 2025 [53]
	Scheduling flexibility and improved session adherence	Easier scheduling, better compatibility with work and family responsibilities, and reduced disruptions to daily routines.	“Personally, I could never attend in-person sessions because of overlaps with worktime, and so I accepted in the moment they told me about the telerehab. It was compatible with my job and unfolded nicely”.	Aliaga-Castillo et al., 2022 [32]
			“I’m busy all the time, so I was sort of trying to get it in, so I was glad it was over. But if it was less time at home, maybe not as many days, just say once a week, doing that [would be better].”	Thomas et al., 2018 [54]
			“It takes less out of our day. We have other children and we both work, and we’re pretty busy so to be able to say “we’re off to speech therapy now” and it’s just one hour out of your day rather than three hours, was great”.	Thomas et al., 2018 [54]
	Rural and remote access	Improved access for rural, remote, or mobility limited populations	“Accessibility for me. Being able to do it in the morning pretty much from home in the space that I’ve set aside. The benefit is that it’s less travel”	Brown et al., 2023 [36]
			“I didn’t have to go someplace else and park and you know, the whole thing”	Wiley et al., 2024 [55]
			“I’d say that it’s a really good medium and it certainly worked well for me because, I can’t travel to Melbourne, so that’s important.”	Lee et al., 2022 [50]
Theme 2: Perceived Efficiency and Continuity of Care	Perceived efficiency	Mixed perceptions of telehealth efficiency, highlighting both the convenience and limitations compared to in-person sessions.	“I’m fairly certain that at least twice, on two occasions certainly if he would have come, it would have been a plus. Well, maybe psychologically, I think, thinking that he could have manipulated your knee, to see in a tangible manner and be able to manipulate it, but hum… it’s the suggestion that I would give, to at least meet, I don’t know how often (…).”	Kairy et al. 2013 [47]
			“Look, I found it very personable, it wasn’t like it was any different really than being in the same room with someone.” (Parent)	Johnsson et al., 2019 [46]
			“I’m probably a bit biased in a way, I sort of feel that physio is such, what would I say, physio is a doing type of thing, to a large extent, I don’t think it’s suitable for telehealth. If you want to have good sessions, I think it has to be face to face.”	Lee et al., 2022 [50]
			“I used to get tired quickly during the day, but after the exercises, I didn’t feel exhausted.”	Erden Guner et al., 2025 [41]
			“If asked ‘would you like to try again,’ I would even though, I mightn’t be fit enough to complete all the exercises, but I still would because it was enjoyable.”	O’Shea et al., 2022 [51]
	Autonomy and control	Greater control over treatment plans and enhanced self-management	“Being able to view my own data and be ‘the captain’ of my own rehabilitation is important to me.”	Dinesen et al., 2019 [40]
			“But of course, you know, with any exercise you get lazy, but this kept us on track… We had to decide when we were going to do our homework [reference to self-management exercise sessions]. But we always did them as much as we were supposed to.”	Wiley et al., 2024 [55]
			“The Teledialog project has provided the framework for individualized rehabilitation at a distance and stimulated me to do activities that I felt were useful for me in my situation.”	Dinesen et al., 2019 [40]
			“Telerehabilitation allows access to healthcare and continuity of care, contributing to self-management of disease conditions in older people.”	Reis et al., 2025 [53]
	Continuity of care	Ongoing support through telehealth, especially during periods when in-person care was unavailable	“Well, I was lucky in this sense because my boss allowed me an hour. So I went into a private room, and I could do the exercise.”	O’Shea et al., 2022 [51]
			“We talk about a pandemic because it was during the pandemic […] I couldn’t go to the clinics because they refused due to the virus. I was asked if I wanted to do it via Zoom. Given the COVID situation, I said yes”	Ouédraogo et al., 2024 [52]
			“I feel I’m not being left alone […] throughout both the social upheaval [in late 2019] and now the pandemic. I have had my therapy uninterruptedly, although virtual, personally I haven’t felt abandoned.”	Aliaga-Castillo et al., 2022 [32]
			“Not having to go to hospital. For those who can’t travel, rehabilitation at home is ideal. And since it’s possible, why not continue.”	Reis et al., 2025 [53]
	Integration into daily routines; overcoming Motivation challenges	Therapy embedded into home and daily life	“If asked “would you like to try again,” I would even though I mightn’t be fit enough to complete all the exercises, but I still would because it was enjoyable.”	O’Shea et al., 2022 [51]
			“…doing it from home isn’t very motivating… It’s a lot harder to concentrate and a lot harder to… get into it”	Hudon et al., 2024 [44]
			“I would jump at the chance to do video conferencing again.”	Thomas et al., 2018 [54]
			“It took away the stress of being in a different environment, it puts me in my own environment. There, I could rest as I wanted, I could… I was at home! it says, ‘There’s no place like home’”	Ouédraogo et al., 2024 [52]
Theme 3: Therapeutic Relationship and Social Presence	Perceived quality of therapeutic alliance	Therapeutic alliance, trust and emotional support	“…we talked about fishing, we talked about hunting, (…) we talked about skiing, hum, of all sorts of things, while I was doing my exercises, we talked about anything and we always had something to say. I think that she knew my whole life” (laughter)	Kairy et al. 2013 [47]
			“it’s helped school to know where we’re at with therapy… [the tele-therapist] was able to contribute and make suggestions and put some strategies in place to help with different issues.”	Johnsson et al., 2019 [46]
			“Some stranger popped up and Zoom and, I’d be like, eugh, I wouldn’t trust them at all.”	Daly et al., 2022 [37]
			“She [PT] engaged me and in a way that, you know, helped me improve my mobility”.	Wiley et al., 2024 [55]
			“It was good, just the hug was missing! The hug twice a week.”	Barboza et al., 2024 [34]
			“I felt more connected to the providers I saw in person than anyone I saw virtually. And even if I saw them [visually] virtually it didn’t have that same feel to it”.	Brehon et al., 2026 [35]
	Communication quality	Clear explanations; improved discussion and feedback	“…because instead of just showing, you have to explain over Telehealth, and that the explanations sometimes take up a lot more time when it’s difficult to explain or, like, to point on your back where it is. It’s easier to show in-person, which helps to kinda save time…”	Hudon et al., 2024 [44]
			“And I really have to be a little bit more pointed and, like, almost talk to her as if I was talking to an [occupational therapy] student, explaining certain clinical observations, but in terms that necessarily don’t have to be, like, professional terms. Like, more understandable terms.”	Angell et al., 2024 [33]
			“The physiotherapist in the videos demonstrated the exercises slowly and explained things easily. I was really surprised how the residents were able to follow everything without any help.”	Dawson et al., 2024 [38]
			“…and because it’s written down there [reference to number of repetitions for each exercise], I found that good, and easy to follow”.	Wiley et al., 2024 [55]
			“I mean I understand what they’re trying to do, but I really don’t understand how you can get them saying, you know, “darfebee” and for that to affect other words.”	Thomas et al., 2018 [54]
			“There was a clear goal, and the physiotherapist really put effort into helping us achieve it. They managed to strike a perfect balance-not too strict, not too relaxed, which is tough to do.”	Erden Guner et al., 2025 [41]
	Emotional connection and trust	Development of rapport and confidence over time	“It was good, just the hug was missing! The hug twice a week.”	Barboza et al., 2024 [34]
			“I’ve known my doctor for over ten years so it’s pretty good rapport, easy prescriptions.”	Dean et al., 2024 [39]
			“Yeah. I think that’s always going to be just the interpersonal barrier, so the best way to build up rapport in a relationship is probably face to face.”	Brown et al., 2023 [36]
			“You know that touch that we need to receive sometimes? So I think that’s what was missing. (…) It’s the contact, because we stay here alone, so it feels a little cold, you know. It’s not easy without human contact.”	Barboza et al., 2024 [34]
			“Initial thoughts of it feeling like just talking over a computer, ‘that wasn’t the case at all’”	Thomas et al., 2018 [54]
			“When I first joined the program, I didn’t know many of the exercises and didn’t speak much. But my confidence grew as I started talking more and getting encouragement.”	Erden Guner et al., 2025 [41]
	Family/caregiver involvement	Caregivers more involved in sessions conducted at home	“My husband also (…) wants to do it sometimes. We have a lot of fun doing it, so it’s like family therapy.”	Barboza et al., 2024 [34]
			“It was very complicated because I didn’t have my children around to help me with the new technology.”	Reis et al., 2025 [53]
			“I appreciate it when my husband keeps pushing and motivating me to keep training and following my goals for my rehabilitation”	Dinesen et al., 2019 [40]
			“Because, like, you know, I felt like if I were to be around them, I probably would have been a distraction. Or, you know, it was just like the whole one-on-one thing, you know”	Angell et al., 2024 [33]
			“My daughter helps me. I don’t use [smartphones or computers] because I don’t know (…) She helps me. She is always by my side.”	Barboza et al., 2024 [34]
Theme 4: Digital and Contextual Challenges (cross-cutting)	Digital challenges	Technological Barriers and Enablers in Telehealth	“a few rough days there that third week where the connection wasn’t fantastic, it kept pausing.”	Thomas et al., 2018 [54]
			“Once my computer kept blacking out and I had to keep coming, leaving, you know, things like that… but I could just trot over here and do my button here [indicates Zoom© icon] and it would come back on… But that was the only glitch”.	Wiley et al., 2024 [55]
			“For me, it was good because my internet is good. So there was no problem. But my colleagues had problems with the internet [connection]”	Barboza et al., 2024 [34]
			“Zoom worked well when we connected the iPad to the TV, we were able to turn the volume of the TV up so the resident could hear better. It also gives a bigger picture as well, so they can see the physio better.”	Dawson et al., 2024 [38]
			“I’m concerned about privacy, of course right. Especially knowing that even Zoom had its own privacy and confidential issues. So, I think maintaining privacy and confidential information is really important-I hope they are doing their due diligence in order to keep our information private.”	Dean et al., 2024 [39]
	Contextual challenges	Difficulty assessing or correcting physical movements	“In terms of physiotherapy, not having someone walking beside you and making corrections as you go along […] it was less, I felt like there was less potential for feedback in terms of physiotherapy”	Ouédraogo et al., 2024 [52]
			“Without being in physical contact, she wasn’t able to do what I really get benefit from, which is, she checks my lungs with the stethoscope and that’s not something you can do remotely.”	Lee et al., 2022 [50]
			“The disadvantage I see is that physical assessment isn’t fully done, the one that involves touch, palpation and actually see in detail if I’m doing my exercise correctly. I don’t know, I might be wrong, but that’s what I feel with this modality.”	Aliaga-Castillo et al., 2022 [32]
			“Personally, I don’t think there is anything that you or I or anyone can do to replace that human contact, that face-to-face contact. It’s a very different interaction between screens, it’s not human.”	Daly et al., 2022 [37]
			“Through teleconsultation, nurses can only see the patient’s face…. But when assessing a stroke patient, being able to observe their movements and body language is crucial.”	Wong AK et al., 2025 [56]
			“After the severe filtering situation, it became difficult to observe physical disabilities, because it was not possible to make a video call at all, unless you used special applications, which unfortunately did not have enough facilities for our work.”	Aarabi MA et al., 2025 [31]
	Digital literacy issues	Challenges using technology especially among some users	“Obviously they would need to be confident in the software themselves, to be able to advise people having difficulties because sometimes these things do drop out and they can be a real impediment to relationship if it’s just frustrating.”	Lee et al., 2022 [50]
			“I’m technophobic—my daughter did a lot of it for me at the beginning”	Wiley et al., 2024 [55]
			“For me, everything was complex […], an elderly person who is not accustomed to technology […] I think it would be challenging”	Ouédraogo et al., 2024 [52]
			“I think if technology is not something you are not comfortable with—I think it increases anxiety trying to figure out how to connect and how to do that. I am fortunate that I have strong Wi-Fi, I have the skills to be able to access it. I have the funds to be able to have a laptop all of those things. So, it widens the gap between those that have [the] resources and those that don’t.”	Dean et al., 2024 [39]
			“I have an IT background in the past… and so I’m used to new applications…. I figured the worst that could happen would be I get disconnected and I would just rejoin the call”	Kringle et al., 2023 [48]
	Home environment constraints	Distractions; limited space or equipment	“The equipment we usually use, either we don’t find them or are very costly.”	Aliaga-Castillo et al., 2022 [32]
			“I think it’s just distraction. With my husband in the office and then with […]”	Kwok et al., 2022 [49]
			“I’m kind of limited with the equipment that I have at home, it is somewhat limiting in what I can do with the exercise program that I’ve got because I’m relying a lot on the equipment that I can use and stuff that I’ve got access to, which is a lot less than what would be available compared to the clinic.”	Brown et al., 2023 [36]
			“lack of rehabilitation equipment at home”	Aarabi MA et al., 2025 [31]
